# miR-155 Knockdown Protects against Cerebral Ischemia and Reperfusion Injury by Targeting MafB

**DOI:** 10.1155/2020/6458204

**Published:** 2020-01-21

**Authors:** Li Zhang, Chao Liu, Chao Huang, Xiaohui Xu, Junfang Teng

**Affiliations:** ^1^Department of Neurology, The First Affiliated Hospital of Zhengzhou University, Zhengzhou, Henan, China; ^2^Department of Neurology, Luoyang Central Hospital Affiliated to Zhengzhou University, Luoyang, Henan, China

## Abstract

**Background:**

Studies have elucidated that the variable expression levels of miRNAs influence the inflammatory process in ischemic stroke. Nevertheless, the impact and potential mechanism of miR-155 in cerebral ischemia-reperfusion injury (CIRI) keep to be incompletely known.

**Methods:**

The levels of miR-155 and MafB were determined via qRT-PCR, western blot, or immunohistochemistry assays in plasma of patients with CIRI, oxygen glucose deprivation/reoxygenation (OGD/R) induced SH-SY5Y cells, and mouse models with middle cerebral artery occlusion (MCAO). The association between miR-155 and MafB was validated via dual-luciferase reporter and western blot assays. Cell viability, apoptosis, invasion, and migration were evaluated through MTT, flow cytometry, Transwell and wound healing assays. Infarction volume was measured in MCAO mouse brain tissues by TTC assay. The expression of inflammatory mediators was measured by ELISA in cells and brain tissues.

**Results:**

miR-155 level was upregulated whereas MafB was downregulated in the plasma of patients with CIRI, OGD/R-induced SH-SY5Y cells, also as mouse models with MCAO injury. Mechanistically, miR-155 directly targeted 3'UTR of MafB and restrained MafB expression in OGD/R injury SH-SY5Y cells. Downregulation of miR-155 attenuated OGD/R-induced injury through increasing proliferation, inhibiting apoptosis, enhancing invasion and migration abilities, and constraining the expression of inflammatory mediators (IL-1*β*, IL-6, and TNF-*α*) and inflammatory enzymes (iNOS and COX-2) in SH-SY5Y cells following OGD/R, while MafB inhibition reversed the protective effects. *In vivo*, downregulating miR-155 reduced the infarction volume in the MACO mouse brain. Furthermore, miR-155 knockdown inhibited the IL-1*β*, IL-6, and TNF-*α*) and inflammatory enzymes (iNOS and COX-2) in SH-SY5Y cells following OGD/R, while MafB inhibition reversed the protective effects.

**Conclusion:**

Our results suggest that miR-155 knockdown alleviated ischemia-reperfusion injury by targeting MafB to improve the neurological function and inhibit inflammation response, highlighting a novel therapeutic strategist for CIRI.

## 1. Introduction

Ischemic cerebral stroke is an acute disease threatening human health, which is caused by the local brain blood supply obstacles and is influenced by multiple pathophysiological process factors, with the characteristics of high morbidity and high fatality [1]. It is generally believed that achieving early blood flow in the ischemic area is an effective method for treatment for such patients. However, this treatment has obvious limitations: on the one hand, it can realize ischemic area blood reperfusion, restore the function of the ischemic area, and alleviate the disease; on the other hand, it is easy to cause cerebral ischemia-reperfusion (CIR) injury, making the condition even worse [2, 3]. The mechanism of CIR is complex, and CIR is regulated by multiple pathways. Studies have shown that oxidative stress, calcium overload, abnormal energy metabolism, immune regulation, inflammatory reaction, and apoptosis are implicated into the pathophysiology of CIR injury, in which inflammatory response and apoptosis are the main factors [4, 5]. Roqué et al. [6] have demonstrated that excessive proinflammatory cytokines and cytotoxic factors, for instance, tumor necrosis factor-*α* (TNF-*α*), interleukin-1*β* (IL-1*β*), and nitric oxide (NO), are secreted in microglia with I/R injury and then aggravate the neuronal damage, which is consistent with the study of Fumagalli et al. [7]. Thereby, inhibiting the excessive reaction of the inflammatory reaction is probably a remedy target to moderate deterioration of ischemic stroke.

MicroRNAs are of length of about 22–25 nucleotides, highly conserved endogenous single-stranded, noncoding small RNAs, which usually bind to the 3′-untranslated region of target mRNAs to inform silenced complex, resulting in transcriptional suppression and degradation of the target RNA and are closely related with the occurrence and development of many diseases [8, 9]. Some studies have also demonstrated that miRNAs also participate in the adjustment of brain damage, myocardial ischemia [10], liver ischemia [11], renal ischemia [12], and other aspects of diseases. Jeyaseelan et al. [13] have used gene chip technology to detect miRNA expression in cerebral ischemia injury rats and have found that more than 30 kinds of miRNAs expressions change significantly and that these miRNAs may participate in the neuropathy and dysfunction process following CIR. Studies by Chu et al. [14] have shown that miR-146a has a positive effect on the mediation of CIR induced inflammation via producing and secreting proinflammatory cytokines.

miR-155 is a typical multifunctional gene revealed in recent years, which is located in the third exon of the B-cell integration cluster (Bic) gene on human monosomy 21. miR-155 is mainly derived from highly expressed noncoding transcription products of activated lymphocytes and mononuclear macrophage and acts as a typical multifunctional gene involved in various pathophysiological processes [15]. Numerous research has identified that miR-155 is a wide range of inflammatory factor and participates in the multiregulation of immune and inflammatory diseases [16]. Hunsberger et al. [17] have demonstrated that miR-155 is obviously upregulated in the cerebral cortex after 24 hours of focal cerebral ischemia. Liu et al. [18] have found that miR-155 expresses more than two-fold changes in brain tissues in the presence of multiple brain lesions, suggesting that miR-155 is an essential factor in brain injury. Therefore, the effects of miR-155 are sustained to be proinflammatory and the inhibition of miR-155 possibly is a novelty target for the therapeutics of cerebral ischemic injury.

MafB is a kind of the large musculoaponeurotic fibrosarcoma oncogene (Maf) family proteins with a basic leucine zipper structure, which acts as a critical transcriptional activator of anti-inflammatory cytokine genes [19]. Considering information about the underlying mechanism of miR-155 in CIR injury is rarely known, we conducted this research to examine the expression of miR-155 and MafB and their relationship in patients with cerebral ischemia-reperfusion injury (CIRI), exploring their biological functions and molecular mechanism in oxygen glucose deprivation/reoxygenation (OGD/R) induced SH-SY5Y cells *in vitro*, as well as in the mouse models with middle cerebral artery occlusion (MCAO) *in vivo*. Our study is probably to define a new perspective for the therapy of CIR injury.

## 2. Materials and Methods

### 2.1. Human Peripheral Blood Sample Collection

A total of 20 patients with CIRI whose diagnoses were confirmed clinically and radiologically by two experienced neurologists from the First Affiliated Hospital of Zhengzhou University from November 2017 to July 2018. Blood specimen (5 ml) was gathered into ethylenediaminetetraacetic acid (EDTA) tubes and centrifuged at 2000 ×g for 15 min at 4°C. Then the upper layer plasma was used to detect the miR-155 and MafB levels of patients with CIRI and healthy subjects via qRT-PCR. The collection of human plasma samples for research purposes was allowed by the Institutional Review Board and the Ethics Committee of the First Affiliated Hospital of Zhengzhou University, and each participant or their legal representatives subscribed to the informed consent form for the research.

### 2.2. Cell Culture

SH-SY5Y cells and 293T cells were provided by American Type Culture Collection (ATCC, USA) and incubated in Dulbecco's modified Eagle medium (DMEM) (Sigma-Aldrich, Grand Island, NY, USA) supplemented with 10% (v/v) fetal bovine serum (FBS; Thermo Scientific, Waltham, MA, USA), 2 mM glutamine (Invitrogen, USA), 100 mg/ml streptomycin, and 100 U/ml penicillin (GIBCO, USA). Cells were cultured in a humid atmosphere containing 5% CO_2_ at 37°C.

### 2.3. OGD/R Models

OGD treatment was conducted when the SH-SY5Y cell density was approximately 80% confluence. Firstly, SH-SY5Y cells were cultured with deoxygenated glucose-free Hanks' Balanced Salt Solution (Invitrogen) and cultured in an anaerobic chamber (Thermo) including 5% CO_2_ and 95% N_2_ at 37°C for 6 h. Afterwards, the medium was changed to standard DMEM medium with 10% FBS and incubated under normoxic environments containing 95% air and 5% CO_2_ for another 24 h at 37°C to reoxygenation. Cells incubated in normal medium and normal environments were used as control.

### 2.4. Dual-Luciferase Reporter Assay

The 3′-UTR fragments from MafB including the supposed wild-type miR-155 binding sites, named MafB-3′-UTR as well as the corresponding mutants (MafB-3′-UTRM) were designed and synthesized by the Shanghai Genomics Institute BGI (Shanghai, China). These constructs were amplified and intromitted into pmir-GLO dual-luciferase reporter vector (Sangon Biotech, Shanghai, China) to generate pmiR-GLO-MafB-3′UTR and pmiR-GLO-MafB-3′UTRM luciferase reporter systems. 293T cells were incubated in 24-well plates and cotransfected with pmiR-GLO-MafB-3′UTR or pmiR-GLO-MafB-3′UTRM and miR-155 or miR-Con using Lipofectamine 2000 (Invitrogen). After culturing for 48 h, we collected the cells and detected the luciferase activity with a Dual-Luciferase® Reporter Assay System (TransGen Biotech, Beijing, China) according to the standard protocols and the associate luciferase activity was evaluated as the ratio of firefly luminescence to Renilla luminescence.

### 2.5. Cell Viability Assay

1 × 10^4^ SH-SY5Y cells/well were cultured at a density of 1 × 10^4^ cells into 96-well microplates for 24 h and then subjected to OGD for 6 h and reoxygenation for 24 h. Subsequently, the treated cells were stained with 10 *μ*l MTT (5 mg/ml) (Sigma, Glpbio, CA, USA) and hatched sequentially at 37°C for another 4 h. Next, the violet formazan crystals were melted fully with an additional 150 *μ*l dimethylsulfoxide (DMSO) then oscillated for 10 min. Subsequently, the optical density (OD) was done in a microplate reader (Thermo, Multiskan FC, USA) at 490 nm.

### 2.6. Apoptosis Analysis

Detection of apoptosis in SH-SY5Y cells was conducted using Annexin V-PE/7-ADD apoptosis detection kit (BD Biosciences, USA) by flow cytometry. The SH-SY5Y cells with the indicated treatment were collected and stained with 10 *μ*l Annexin V-PE and 5 *μ*l 7-ADD in 200 *μ*l binding buffer for 20 min at room temperature in the dark. Eventually, total cell apoptosis was determined by a Beckman Coulter flow cytometer (Beckman USA) and the data were calculated with EXPO32 ADC software (Beckman).

### 2.7. Cell Invasion Assay

The cell invasion ability was detected using polycarbonate Membrane (6.5 mm × 8 *μ*m pore size) and Transwell Inserts (Corning Costar, NY). The upper chamber was covered with OGD/R-treated SH-SY5Y cells (2 × 10^4^ cells/well) with indicated transfection suspending in serum-free medium, and the lower chamber was covered with a complete medium as a chemoattractant. After hatching for 48 h at 37°C and 5% CO_2_, the noninvaded cells on the upper chamber were mechanically wiped off. The invaded cells were washed, immobilized in 4% paraformaldehyde for 20 min, dyed by 0.1% crystal violet for 10 min (Beyotime, Wuhan, China), and quantified in at 200x magnification under the Olympus inverted microscope (Olympus, Tokyo, Japan).

### 2.8. Wound Healing Assay

The cells (2 × 10^5^/ml) with indicated treatments were seeded into a six-well plate and a scratch was made by utilizing a 100 *μ*l tip until the cells formed a confluent monolayer. After 48 h of culture, the closure of scratch was viewed and imaged under the inverted microscope (40x magnification). The percentage of the total cell-free zones was measured to appraise the cells migration capacity using ImageJ (National Institutes of Health).

### 2.9. Animals and Treatments

Male C57BL/6J mice at the age of 6 weeks (22–25 g) were supplied by HFK Bioscience Company (Beijing, China) and housed and maintained in a standard environment allowing open access to food and water. 36 mice were distributed into four groups (*n* = 9 per group): sham, MCAO + saline, MCAO + miR-155, and MCAO + anti-miR-155. miR-155 mimics (miR-155) and miR-155 antagomir (anti-miR-155) were injected into mice brain tissues via right intracerebroventricular injection. After 24 h injection, MCAO operation was performed on mice following previously reported procedures [20]. Mice were initially anesthetized with pentobarbital sodium (50 mg/kg i.p.) (Sigma). Then, the whole operation process on mice was conducted with sustaining at 37°C on the heating plate. Subsequently, a midline incision was made to reveal the right common carotid artery (CCA), external carotid artery (ECA), and internal carotid artery (ICA) under an operating microscope. MCAO was established via inserting a surgical nylon monofilament (0.18 mm) lightly from the right ECA into the ICA to block the origin middle cerebral artery in the back of blocked CCA using a heparin-dampened nylon suture. After MCAO for 60 min, the monofilament was taken out and the clamp on CCA was removed to let reperfusion for another 24 h. The sham animals were subjected to identical treatments without occlusion as controls. All mice experiments were carried out strictly in accordance with the Animal Protection Legislation and approved by the Ethics Committee of the Institutional Animal Care and Use Committee of Zhengzhou University.

### 2.10. Staining with 2-3-5 Triphenyltetrazolium Chloride (TTC)

At 24 h following MCAO, five mice in each group were deeply tranced with intraperitoneal pentobarbital and the brains were promptly separated and frozen in −20°C for 30 min. Subsequently, the tissues were cut coronally sliced into 2 mm thick sections, which were then stained by 10 ml of 2% 2,3,5-tripenyltetrazolium chloride (TTC, Sigma) incubated for 10 min at 37°C in the dark and subsequently immobilized with 4% paraformaldehyde for 24 h. The infarct volume was represented by white coloring, while the normal brain area appeared red. TTC-colored sections were photographed under a microscope. Cerebral infarction volume was measured using Image J, and the calculation formula was as follows: percentage of infarction volume = infarction volume/total volume × 100%.

### 2.11. Immunohistochemistry Staining Assay

The expression of MafB in brain tissues of MACO mouse was evaluated by immunohistochemical staining. The brain tissues were immobilized in 4% paraformaldehyde, inserted into paraffin, and then sliced in 3 *μ*m sections. The primary antibody against MafB (Abcam, Cambridge, MA, USA) was used at a dilution of 1 : 500 and incubated at 4°C overnight and then incubated with the matching secondary HRP-conjugated antibodies at room temperature for 1 h. Finally, the sections were counterstained with hematoxylin reagent (Shanghai Honsun Biological Co., Ltd. Shanghai, China) and the staining intensity was visualized under a microscope.

### 2.12. Enzyme-Linked Immunosorbent Assay (ELISA)

To further validate the alteration of inflammatory cytokines (IL-1*β*, IL-6, and TNF-*α*) release in cell-free supernatants and the brain tissue homogenates in response to the indicated treatments, ELISA was performed using the ELISA kit (Abcam, Cambridge, MA, USA) in accordance with the standard methods. The absorbance was detected using a microplate reader at 450 nm.

### 2.13. RNA Isolation and Reverse Transcription Quantitative Polymerase Chain Reaction (qRT-PCR)

Total RNA samples and miRNAs of human plasma, SH-SY5Y cells, and brain tissues were extracted by TRIzol reagent (Life Technologies, Rainbio, MA, USA) and miRNeasy Mini Kit (Qiagen) in accordance with the manufacturer's manual, respectively. The purity of RNA was checked at the absorbance of 260/280 and 260/230 with the Ultra-micro UV Spectrophotometer (Beckman Coulter, CA, USA). For cDNA synthesis of MafB, iNOS, and COX-2, 1 *μ*g total RNAs was reverse-transcribed using the EasyScript First-Strand cDNA Synthesis SuperMix (TransGen Biotech Co., Ltd., Beijing, China) and the RT-PCR was performed via SYBR Premix Ex Taq (TaKaRa, Shiga, Japan). For detection of miR-155, cDNA was constructed using the TaqMan MicroRNA Reverse Transcription Kit (Applied Biosystems, CA, USA), and RT-PCR reactions were implemented via the Express SYBR GreenER miRNA qRT-PCR kit (Invitrogen, Rainbio, MA, USA). All amplification assays were performed on a Bio-Rad Real-Time PCR equipment (Bio-Rad, Hercules, CA) based on the standard protocol. The expression levels of MafB, iNOS, and COX-2 were standardized to GAPDH, and miR-155 was normalized against *U6* expression. The correlative quantification analysis of target genes was detected by comparing to the internal reference using formula 2^−∆∆Ct^, where ∆Ct = Ct_miR−XorX_ − Ct_U6 or GAPDH_.

### 2.14. Western Blot Analysis

The proteins of cerebral tissues and cells were isolated and harvested by ice-cold RIPA lysis buffer (Sigma) supplemented with protease inhibitors (Thermo Fisher) and quantified by BCA assay (Beyotime, Biotechnology, Nanjing, China) according to the standard protocols. Equal amounts of protein lysates of each sample were fractionated on 10% SDS-PAGE gels and subsequently transferred onto the polyvinylidene difluoride membranes (Millipore, Bedford, USA). Membranes were blocked for 1 h in 5% skim milk containing Tris-buffered saline (pH 7.4) and 0.1% Tween 20 at room temperature. Membranes were then hatched with primary antibodies at 4°C overnight and respective secondary antibodies at room temperature for 2 h. The primary antibodies anti-MafB, anti-iNOS, anti-COX-2, anti-*β*-actin, and the HRP-conjugated secondary antibodies were all purchased from Abcam. Finally, the immunoreactive bands were visualized using ECL detection reagents (Bio-Rad, Amersham, Sweden) with the chemiluminescence detection system (Thermo, Bethesda, MD, USA). *β*-actin served as the for normalization control.

### 2.15. Statistical Analysis

Data were demonstrated as mean ± SD from at least three independent experiments. Statistics and graphs analyses were conducted using SPSS 21.0 Software and GraphPad Prism 5.0 Software. Differences between the two samples were detected via the Student's *t* test, and multiple comparisons were implemented by One-Way ANOVA test. *P* value <0.05 was considered significant.

## 3. Results

### 3.1. Expression of miR-155 and MafB in Ischemic Stroke Patients

**Figure 1:**
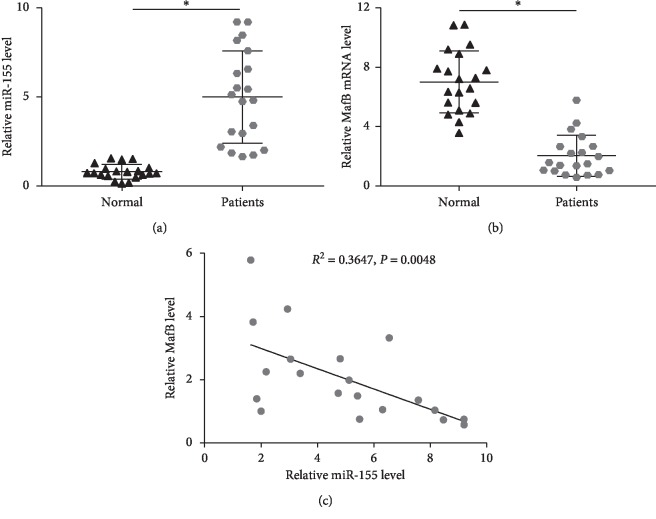
The expression of miR-155 and MafB in patients with CIRI. qRT-PCR was implemented to investigate the plasma levels of miR-155 (a) and MafB (b) in patients with CIRI and controls. (c). Correlation between miR-155 and MafB levels in plasma ^*∗*^*P* < 0.05.

We primarily detected the expression patterns of miR-155 and MafB in plasma of 20 patients with CIRI using qRT-PCR. Compared to the control, miR-155 was remarkably enhanced in patients with CIRI (Figure 1(a)). However, the expression of MafB was evidently attenuated in CIRI patients by comparison to the healthy subjects (Figure 1(b)). Furthermore, correlation analysis demonstrated that miR-155 level was negatively associated with MafB level in patients with CIRI (*R*^2^ = 0.3647, *P*=0.0048, Figure 1(c)).

### 3.2. Expression of miR-155 and MafB in OGD/R SH-SY5Y Cells

**Figure 2:**
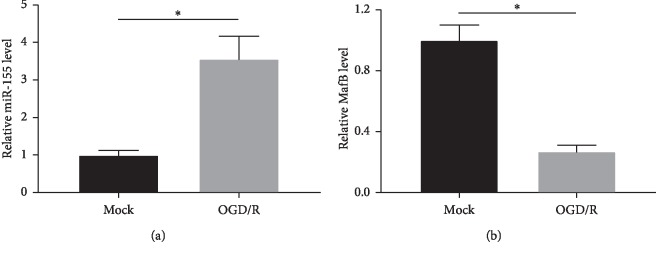
The expression of miR-155 and MafB in OGD/R-treated SH-SY5Y cells. (a) The quantitative analysis of miR-155 expression in OGD/R-induced SH-SY5Y cells by qRT-PCR analysis. (b) The expression of MafB in OGD/R-treated SH-SY5Y cells through qRT-PCR analysis ^*∗*^*P* < 0.05.

Further, we examined the expression patterns of miR-155 and MafB in CIR models *in vitro* via qRT-PCR. As displayed in Figure 2(a), the treatment of OGD/R induced the expression of miR-155 in SH-SY5Y cells compared with the control group. Additionally, the qRT-PCR results demonstrated that MafB level was dramatically decreased following OGD/R injury in comparison to the control (Figure 2(b)). The results above revealed that miR-155 and MafB played a crucial role in SH-SY5Y cells with OGD/R injury.

### 3.3. MafB Is a Direct Target Gene of miR-155

**Figure 3:**
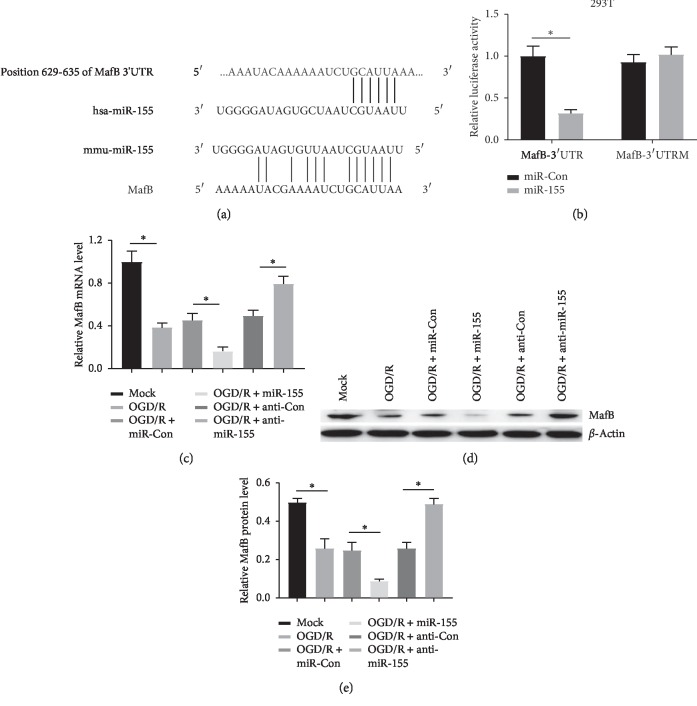
miR-155 directly targeted on MafB. (a) The target site of miR-155 and MafB 3′UTR was measured based on TargetScan and microRNA.org. (b) Investigation of the regulatory effect of miR-155 on MafB expression using dual-luciferase reporter assay in 293T cells. (c) and (d and e) The expression of MafB in OGD/R-induced SH-SY5Y cells transfected with miR-155 or anti-miR-155 was measured through qRT-PCR and western blot analyses respectively ^*∗*^*P* < 0.05.

To further confirm the biology molecular mechanism of miR-155 in OGD/R-treated SH-SY5Y cells, Target Scan (http://www.targetscan.org/) and microRNA.org (http://www.microrna.org/) were used to predict the target genes of miR-155. Prediction outcomes revealed that MafB was the putative target of miR-155 in the genetic systems of human and mice and the binding regions between miR-155 and MafB were exhibited in Figure 3(a). Dual-luciferase reporter assay indicated that cotransfection of the wild-type MafB-3′ UTR with miR-155 remarkably reduced luciferase activity compared with the miR-Con transfection group in 293T cells, while the luciferase activity had no obvious change in MafB-3′ UTRM and miR-155 cotransfected cells, which suggested that miR-155 targets MafB at the predicted binding site (Figure 3(b)) andnd qRT-PCR and western blot results further confirmed that miR-155 overexpression markedly suppressed MafB expression in OGD/R-treated SH-SY5Y cells, while an opposite effect was found in the anti-miR-155 group (Figures 3(c), 3(d), and 3(e)). These results confirmed that miR-155 could negatively regulate MafB via directly binding to the 3′UTR of MafB.

### 3.4. Knockdown of miR-155 Enhanced Viability, Invasion, and Migration of OGD/R-Treated SH-SY5Y Cells and Constrained Apoptosis by Targeting MafB

**Figure 4:**
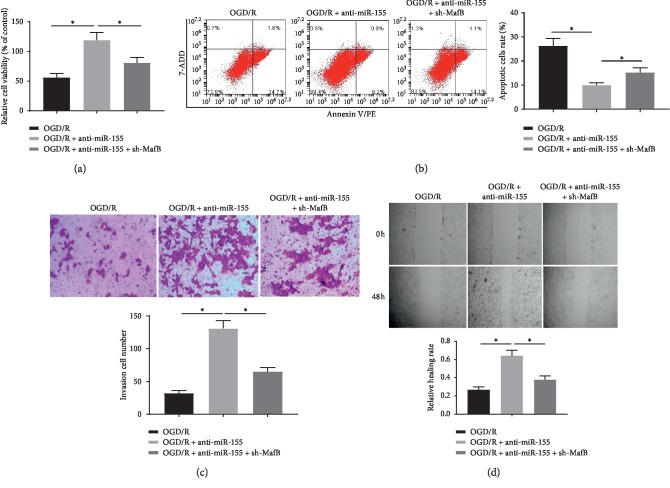
miR-155 downregulation enhanced viability, invasion, and migration of OGD/R-treated SH-SY5Y cells and constrained apoptosis by targeting MafB. (a) Viability of OGD/R-treated SH-SY5Y cells transfected with anti-miR-155 alone or in combination with sh-MafB was measured by MTT assays. (b) The rates of apoptosis in OGD/R-treated SH-SY5Y cells with the indicated treatments were determined by flow cytometry assay at 48 h. (c) The cells invasion ability was determined with Transwell assay at 48 h. (d) The cells migration capacity was detected through wound healing assay at 0 and 48 h ^*∗*^*P* < 0.05.

Subsequently, to corroborate the precise biological function of miR-155 and MafB in SH-SY5Y cells receiving OGD/R treatment, some function experiments of cells were conducted. MTT assay illustrated that suppression of miR-155 obviously enhanced the cell activity of SH-SY5Y cells receiving OGD/R treatment comparing to the control, whereas the sh-MafB reversed the effects (Figure 4(a)). Furthermore, flow cytometry analysis demonstrated that OGD/R-induced cell apoptosis was also dramatically decreased by inhibiting the expression of miR-155. By contrast, these protections were further abrogated when downregulated by MafB expression (Figure 4(b)). As manifested in Figures 4(c) and 4(d), the cell invasion and migration capabilities were increased by downregulation of miR-155; by comparison with only OGD/R treatment, the augment can be reversely modulated via sh-MafB. These results demonstrated that anti-miR-155 attenuated OGD/R-induced injury in SH-SY5Y cells through promoting the viability, decreasing apoptosis, and strengthening the invasion and migration capabilities of cells by regulating MafB.

### 3.5. Knockdown of miR-155 Repressed the Inflammatory Response of OGD/R-Induced SH-SY5Y Cells

**Figure 5:**
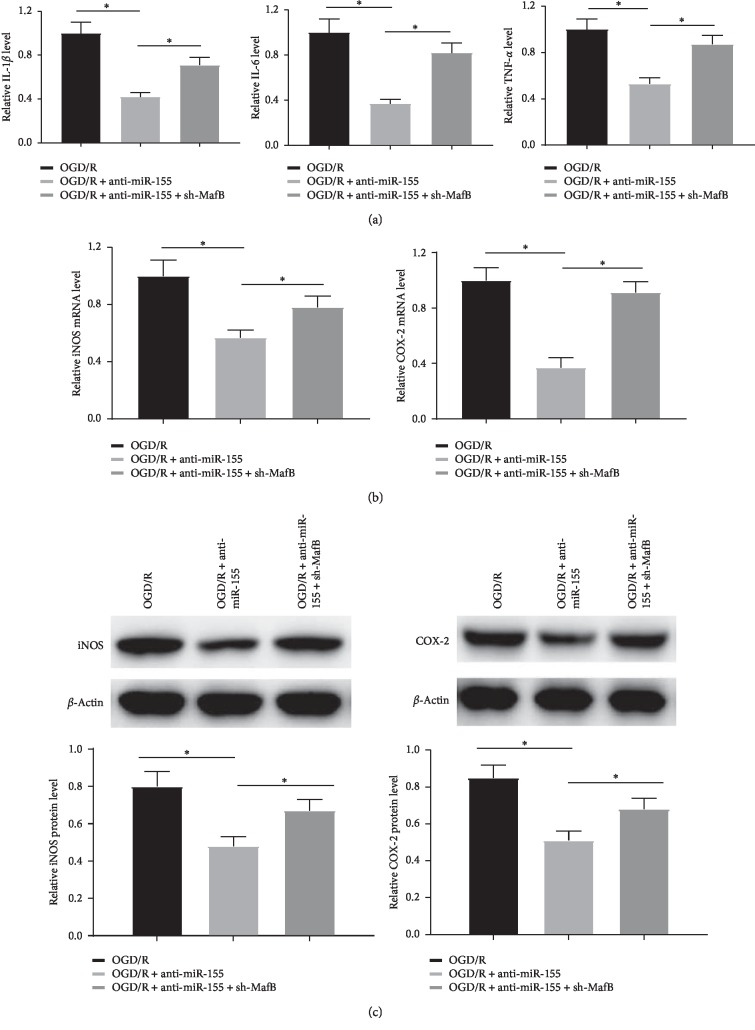
miR-155 downregulation repressed the inflammatory response of OGD/R-induced SH-SY5Y cells. (a) The expression of IL-1*β*, IL-6, and TNF-*α* in OGD/R-induced SH-SY5Y cells transfected with anti-miR-155 alone or in combination with sh-MafB was detected by ELISA assay. (b) and (c) The expression of iNOS and COX-2 was determined via qRT-PCR and western blot analyses, respectively ^*∗*^*P* < 0.05.

Abundant evidence has demonstrated that proinflammatory cytokines are implicated into the pathological process of CIRI [21]. To assess whether miR-155 and MafB participate in ischemia that induces inflammation and injury, the levels of inflammation factors (IL-1*β*, IL-6, and TNF-*α*), which are markers of inflammation, were measured in OGD/R-treated SH-SY5Y cells by ELISA. As demonstrated in Figure 5(a), the results revealed that the expression of IL-1*β*, IL-6, and TNF-*α* was noticeably downregulated by the introduction of anti-miR-155 compared with the OGD/R-treated group, which was further markedly reversed by sh-MafB. Furthermore, the levels of regulated factor iNOS and COX-2 were investigated via qRT-PCR and western blot, which was closely associated with inflammation and acted as inflammatory enzymes, and the outcomes showed that anti-miR-155 abridged the production of iNOS and COX-2 in OGD/R-treated SH-SY5Y cells, which protective effects were further reversed by knockdown of MafB (Figures 5(b) and 5(c)). Collectively, the data indicated that the downregulation of miR-155 decreased the secretion of ischemia-triggered proinflammation factors against the OGD/R damage probably via targeting MafB.

### 3.6. Knockdown of miR-155 Relieved Ischemia Injury in Mouse with MCAO

**Figure 6:**
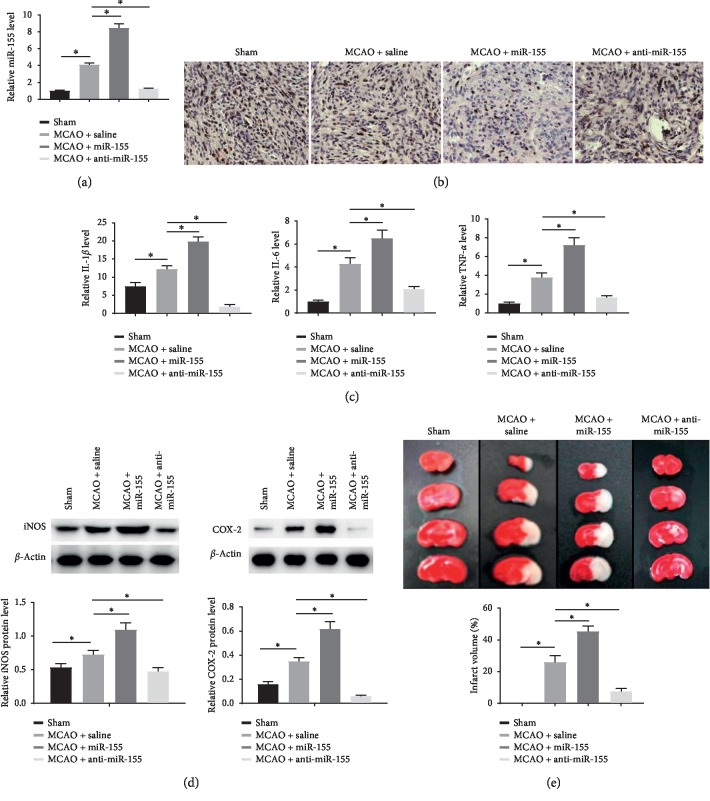
Knockdown of miR-155 relieved ischemia injury in mice with MCAO. (a) miR-155 levels in mouse brain tissues after MCAO by qRT-PCR. (b) MafB protein level in mouse brain tissues after MCAO via immunohistochemistry assay. (c) The expression of IL-1*β*, IL-6, and TNF-*α* in mouse brain tissues was determined by ELISA assay. (d) The levels of iNOS and COX-2 in mouse brain tissues were investigated by western blot. (e) Representative images of TTC-stained coronal sections and analysis of the volumes of infarction in mouse brain tissues ^*∗*^*P* < 0.05.

For the purpose of detecting the underlying mechanism of the effects of miR-155 on ischemia injury *in vivo*, MCAO mouse models were established. qRT-PCR results demonstrated that the level of miR-155 was raised in the MCAO group in comparison to the sham group. Compared to the MCAO group, miR-155 was remarkably enhanced in the miR-155 group and decreased in the anti-miR-155 group (Figure 6(a)). In addition, as shown in Figure 6(b), the protein level of MafB in the cortex of the injury aspect was significantly reduced in the MCAO group compared with the sham group. In comparison to the MCAO group, the level of MafB in the miR-155 group was inhibited, while significantly heightened after treatment with miR-155 inhibitor. Furthermore, ELISA and western blot assays showed that MCAO treatment induced a significant increase in the production of IL-1*β*, IL-6, and TNF-*α* and upregulated the protein levels of iNOS and COX-2 in brain tissues. Moreover, miR-155 overexpression significantly enhanced the levels of IL-1*β*, IL-6, TNF-*α*, iNOS, and COX-2 in MCAO brain tissues, while miR-155 knockdown alleviated the above observations (Figures 6(c) and 6(d)). Furthermore, the brain infarction volumes in the miR-155 group were raised enormously compared to the MCAO group, whereas miR-155 downregulation significantly reduced the infarction volumes induced by MCAO (Figure 6(e)). Taken together, these results further confirmed that miR-155 knockdown could relieve ischemia injury in mice with MCAO.

## 4. Discussion

Cerebral Ischemia-Reperfusion (CIR) injury is a complex systemic process that affects multiple tissues and organs and causes irreversible damage which leads to a cascade of dysfunction and structural damage. In humans, microRNAs account for 2% of the total number of genes and regulate about one-third of gene function participating in important cellular pathways as well as various physiological and pathological processes. Recently, the relation between miRNAs and CIRI has been searched by accumulating researches. For instance, the study of Feng et al. [22] has demonstrated that miR-310a has proinflammatory effects in the progress of CIRI by targeting NGRG2 to induce the inflammatory response and cell apoptosis. Studies have indicated that certain miRNAs in the plasma are not only implicated into the pathophysiological process of CIR but also play a crucial role in clinical diagnosis and intervention treatment as a specific and sensitive indicator of cerebral ischemia [23].

Significant changes have been found in the levels of miR-155 in the hippocampus and blood after cerebral ischemia injury [24]. In hepatic ischemia-reperfusion, the lack of miR-155 can upregulate SOCS-1 expression and reduce the hepatic damage [11]. Maf is a family described as transcriptional activators and MafB plays a crucial part in mediating the inflammatory reaction in immune cells [19]. The results of our study revealed that miR-155 was upregulated while MafB was downregulated in patients with CIRI and CIR cell models *in vitro* and mouse models *in vivo*. An inverse correlation between miR-155 and MafB expression in plasma of patients with CIRI was confirmed. Furthermore, miR-155 directly targeted MafB and inhibited its expression in SH-SY5Y cells.

Multiple studies have testified that microRNAs have been widely associated with the function regulations of organs under ischemia and reperfusion. As in CIR, the dysregulation of miR-320 results in the imbalance of expression level of myocardial protective protein HSP20 and increased apoptosis rates of cardiomyocytes [25]. Studies by Liang et al. have proved that miR-454 can increase colon cancer cells survival, proliferation, and migration ability [26]. Caballero-Garrido et al. [27] have illustrated that intravenous injection of miR-155 inhibitor induces angiogenesis, inhibits brain tissue damage, as well as promotes CIR restoration in MCAO mice. As some studies have reported, ischemia and reperfusion injury can result in reduced cell proliferation, migration, and invasion capabilities and accelerate apoptosis in nerve cells [28, 29], which is identified with our results. Interestingly, our study outcomes revealed that miR-155 suppression played protective effects via promoting OGD/R-induced cells survival ability, migration, invasion capabilities, and mitigating apoptosis and suppressing the infarct area in the MCAO mouse model through negatively regulating MafB expression, which further confirmed that miR-155 and MafB were functional regulators involved in CIR injury.

Increasing evidence suggests that inflammation basis for the pathological development and progression of ischemia-reperfusion injury, which can lead to infiltration of local mononuclear macrophages, promote lipid deposition and lead to early lesions such as brain damage [30]. Therefore, suppressing the inflammatory reaction is probably an available remedial tactic to attenuate ischemia-reperfusion injury. It has been shown that miR-155 is a key mediator for inflammatory regulation and obviously inhibits the production of anti-inflammatory factors [31]. Nazari-Jahantigh et al. [32] have also confirmed that miR-155 can attenuate the signaling of proinflammatory factor NF-κB by directly inhibiting the expression of BCL-6. Tang et al. [11] have identified that miR-155 deficient can upregulate the expression of IL-10 and downregulate the expression of TNF-*α*, IL-6, and IL-12p40 by decreasing ALT levels, so as to regulate the inflammatory reaction and stimulate innate immune to ameliorate liver ischemia. Consistently, our study demonstrated that systematic subjection to miR-155 inhibitor noticeably reduced ischemia-triggered proinflammatory cytokines IL-1*β*, IL-6, and TNF-*α* levels and inflammatory enzymes iNOS and COX-2 levels in CIR injury models *in vitro* and *in vivo*, while MafB inhibition or miR-155 overexpression can obviously reverse the impact of anti-miR-155 on inhibiting inflammation and then further augment CIR injury. Collectively, suppression of miR-155 may play protective roles on inflammation inhibition and neuron impairment via targeting MafB in CIR injury.

## 5. Conclusion

In conclusion, our study confirmed an increased plasma level of miR-155 and reduced MafB in CIRI patients, OGD/R-induced SH-SY5Y cells and MCAO mouse, and miR-155 knockdown promoted neurological biological function and mitigated the inflammation response induced by CIR injury by targeting MafB. Our study suggests that miR-155 and MafB can be developed as novel therapeutic strategist targets in treating neurological injury during CIRI.

## Data Availability

The data used to support the findings of this study are included within the article.

## References

[1] Chugh C. (2019). Acute ischemic stroke: management approach. *Indian Journal of Critical Care Medicine*.

[2] Zhang J.-F., Shi L.-L., Zhang L. (2016). MicroRNA-25 negatively regulates cerebral ischemia/reperfusion injury-induced cell apoptosis through fas/FasL pathway. *Journal of Molecular Neuroscience*.

[3] Csonka C., Sarkozy M., Pipicz M. (2016). Modulation of hypercholesterolemia-induced oxidative/nitrative stress in the heart. *Oxidative Medicine and Cellular Longevity*.

[4] Rana A. K., Singh D. (2018). Targeting glycogen synthase kinase-3 for oxidative stress and neuroinflammation: opportunities, challenges and future directions for cerebral stroke management. *Neuropharmacology*.

[5] Belisário A. R., Silva C. M., Velloso-Rodrigues C., Viana M. B. (2018). Genetic, laboratory and clinical risk factors in the development of overt ischemic stroke in children with sickle cell disease. *Hematology, Transfusion and Cell Therapy*.

[6] Roqué P. J., Dao K., Costa L. G. (2016). Microglia mediate diesel exhaust particle-induced cerebellar neuronal toxicity through neuroinflammatory mechanisms. *Neurotoxicology*.

[7] Fumagalli S., Perego C., Pischiutta F. (2015). The ischemic environment drives microglia and macrophage function. *Frontiers in Neurology*.

[8] Kim B.-K., Kim I., Yoon S. K. (2015). Identification of miR-199a-5p target genes in the skin keratinocyte and their expression in cutaneous squamous cell carcinoma. *Journal of Dermatological Science*.

[9] Ariyoshi J., Momokawa D., Eimori N., Kobori A., Murakami A., Yamayoshi A. (2015). Development of novel antisense oligonucleotides for the functional regulation of RNA-induced silencing complex (RISC) by promoting the release of microRNA from RISC. *Bioconjugate Chemistry*.

[10] Zhu X., Zhao Y., Hou W., Guo L. (2019). MiR-153 regulates cardiomyocyte apoptosis by targeting Nrf2/HO-1 signaling. *Chromosome Research*.

[11] Tang B., Wang Z., Qi G. (2015). MicroRNA-155 deficiency attenuates ischemia-reperfusion injury after liver transplantation in mice. *Transplant International*.

[12] Liu Z., Yang Q., Wei Q., Chang Y., Qu M., Yu L. (2019). The protective effect of miR-377 inhibitor against renal ischemia-reperfusion injury through inhibition of inflammation and oxidative stress via a VEGF-dependent mechanism in mice. *Molecular Immunology*.

[13] Jeyaseelan K., Lim K. Y., Armugam A. (2008). MicroRNA expression in the blood and brain of rats subjected to transient focal ischemia by middle cerebral artery occlusion. *Stroke*.

[14] Chu B., Zhou Y., Zhai H., Li L., Sun L., Li Y. (2018). The role of microRNA-146a in regulating the expression of IRAK1 in cerebral ischemia-reperfusion injury. *Canadian Journal of Physiology and Pharmacology*.

[15] Faraoni I., Antonetti F. R., Cardone J., Bonmassar E. (2009). miR-155 gene: a typical multifunctional microRNA. *Biochim Biophys Acta*.

[16] Lippai D., Bala S., Catalano D., Kodys K., Szabo G. (2014). Micro-RNA-155 deficiency prevents alcohol-induced serum endotoxin increase and small bowel inflammation in mice. *Alcoholism: Clinical and Experimental Research*.

[17] Hunsberger J. G., Fessler E. B., Wang Z., Elkahloun A. G., Chuang D. M. (2012). Post-insult valproic acid-regulated microRNAs: potential targets for cerebral ischemia. *American Journal of Translational Research*.

[18] Liu D.-Z., Tian Y., Ander B. P. (2010). Brain and blood microRNA expression profiling of ischemic stroke, intracerebral hemorrhage, and kainate seizures. *Journal of Cerebral Blood Flow & Metabolism*.

[19] Singh T., Colberg J. K., Sarmiento L. (2019). Loss of MafA and MafB expression promotes islet inflammation. *Scientific Reports*.

[20] Yu Y., Zhang X., Han Z., Zhao W., Zhang L. (2019). Expression and regulation of miR-449a and AREG in cerebral ischemic injury. *Metabolic Brain Disease*.

[21] Elton T. S., Selemon H., Elton S. M., Parinandi N. L. (2013). Regulation of the MIR155 host gene in physiological and pathological processes. *Gene*.

[22] Feng T., Han B.-H., Yang G.-L. (2019). Neuroprotective influence of miR-301a inhibition in experimental cerebral ischemia/reperfusion rat models through targeting NDRG2. *Journal of Molecular Neuroscience*.

[23] Laterza O. F., Lim L., Garrett-Engele P. W. (2009). Plasma MicroRNAs as sensitive and specific biomarkers of tissue injury. *Clinical Chemistry*.

[24] Tili E., Michaille J.-J., Cimino A. (2007). Modulation of miR-155 and miR-125b levels following lipopolysaccharide/TNF-*α* stimulation and their possible roles in regulating the response to endotoxin shock. *The Journal of Immunology*.

[25] Song C.-L., Liu B., Diao H.-Y. (2014). The protective effect of microRNA-320 on left ventricular remodeling after myocardial ischemia-reperfusion injury in the rat model. *International Journal of Molecular Sciences*.

[26] Liang H.-L., Hu A.-P., Li S.-L., Xie J.-P., Ma Q.-Z., Liu J.-Y. (2015). MiR-454 prompts cell proliferation of human colorectal cancer cells by repressing CYLD expression. *Asian Pacific Journal of Cancer Prevention*.

[27] Caballero-Garrido E., Pena-Philippides J. C., Lordkipanidze T. (2015). In vivo inhibition of miR-155 promotes recovery after experimental mouse stroke. *Journal of Neuroscience*.

[28] Xiao B., Chai Y., Lv S. (2017). Endothelial cell-derived exosomes protect SH-SY5Y nerve cells against ischemia/reperfusion injury. *International Journal of Molecular Medicine*.

[29] Tian J., Xu H., Chen G. (2017). Roles of lncRNA UCA1-miR-18a-SOX6 axis in preventing hypoxia injury following cerebral ischemia. *International Journal of Clinical and Experimental Pathology*.

[30] Siniscalchi A., Gallelli L., Malferrari G. (2014). Cerebral stroke injury: the role of cytokines and brain inflammation. *Journal of Basic and Clinical Physiology and Pharmacology*.

[31] Cardoso A. L., Guedes J. R., Pereira de Almeida L., Pedroso de Lima M. C. (2012). miR-155 modulates microglia-mediated immune response by down-regulating SOCS-1 and promoting cytokine and nitric oxide production. *Immunology*.

[32] Nazari-Jahantigh M., Wei Y., Noels H. (2012). MicroRNA-155 promotes atherosclerosis by repressing Bcl6 in macrophages. *Journal of Clinical Investigation*.

